# Notes on Pyorrhea Alveolaris

**Published:** 1899-01

**Authors:** O. N. Heise

**Affiliations:** Cincinnati


### Notes on Pyorrhea Alveolaris.

BY O. N. HEISE, M.D., D.D.S., CINCINNATI.

Read before the Ohio State Dental Society, December, 1898.

"Read many such, and then ask what is to be done."
ROBERT BURTON—*Anatomy of Melancholy.*

It will not be my aim to present for your consideration a long and learned dissertation—"*Quellen-studium,*" as the Germans call it—on the etiology, pathology and scientific treatment of pyorrhea alveolaris, but content myself with offering a few cursory remarks on the subject which is of such paramount importance and practical interest to us all, yet looked upon as a *bete noir.*

To such an extent has this disease become prevalent that at least twenty-five percent (according to Dr. Talbot) of all people over twenty-five years of age are thus more or less afflicted. Twenty-five years ago it was not considered of much importance by the dentist; to-day, it is giving him more trouble than caries of the teeth. If, therefore, I can add anything to assist in bringing about a more careful examination of the mouths of our patients in order to establish an early diagnosis and thereby effect a speedy and permanent cure, I will feel that my labor, however slight, has not been in vain.

It is in the early recognition and treatment of this disease that we can do the most good.

The term Pyorrhea Alveolaris, so universally used, I think, has been a hindrance to the early and correct diagnosis and treatment of the trouble, as many have thereby been led to believe that unless pus is present they are not dealing with this disease. Nothing, in my estimation, is more erroneous; the presence of pus is not a requisite, but an accidental complication due to an infection of the pocket with streptococci and staphylococci. Any number of cases run their course without having or showing that symptom to any degree, or in fact, not at all. It does seem to me that we have looked upon it as a strictly dental disease, all the trouble emanating from the teeth (and the tartar deposits upon the roots), everything seeming to center upon the peridental membrane, leaving out of consideration the influence exerted by the varying conditions of the alveolar process, and the gum tissue surrounding it. The presence of pus and deposits upon the teeth are looked upon as the cause of the disease by a great many; whereas, they are only secondary. In course of time, it is true, they do act as exciting agents in its further development, and as a decided hindrance to the establishment of a cure.

In order to bring out this phase of the subject, I cannot do better than to present for your consideration certain notes on pyorrhea alveolaris, which I presented a year or more ago at a meeting of our City Dental Society comprising some of the results of Prof. Baume's investigation regarding this disease. I do not claim that his ideas are new, or proved beyond a doubt, but in my conversations with dentists about pyorrhea alveolaris, they all seemed to be unfamiliar with these ideas, and I find few if any references to Prof. Baume's work in any of our literature Hence I have deemed it best to again present them in this way.

According to Prof. Baume, pyorrhea alveolaris is an accidental symptom of numerous and varied pathological processes in the bone, periosteum, periodonteum and gingiva, all having the same result, namely, the inducing of an atrophy or absorption of the weak and delicate alveolar lamella, modified by the peculiarities of the anatomical construction of the so affected parts, thin paper like septæ, lax gum tissue, weak development of the roots, and the entrance and deposition of foreign matter, such as mucus, tartar, food, and bacteria into the pockets, thereby presenting a variety of forms of this disease. Prof. Baume suggests the name of "atrophia alveolaris præcox," the wasting of the alveolar ridge and presence of pockets being the main feature of this disease, and not the flow of pus, or the presence of tartar. A premature loosening of the teeth presents itself often without any apparent cause. In the majority of cases we can trace some underlying constitutional trouble, or some local lesion. Of the constitutional causes there are many, such as syphilis, diabetes, tabes, gout, rheumatoid arthritis, digestive disturbances, etc. Among poisons, we have mercury, lead, arsenic, phosphorus, and others. In women the diseases of generative organs, disorders of menstruation, pregnancy, and the climacteric period, are very favorable to the development of gingival irritation. Many affections of the jaw without gingivitis will tend to the premature loosening of the teeth.

As a rule, however, atrophia alveolaris præcox and gingivitis marginalis not only co-exist, but are dependent on one another; and in the majority of cases, it is the gingivitis that acts as the exciting cause. Disturbances of innervation have a special influence in bringing about a premature alveolar atrophy. In tabes dorsalis we have a rapid progressing loosening of the teeth, due to the wasting or dissolution of the alveolar process, brought about by a sclerosis of the trigeminal nerve in its branches or its seat of origin.

Local diseases affecting the nutrient supply of the alveolar lamella and the parts adjoining tend to a change in the density and firmness of the same. Such are diseases of the pericementum, periosteum, and principally of the gingiva.

Examples of the former we have in both acute and chronic pericementitis, and osteitis, where the teeth are loosened to a great extent and may remain so permanently, without the gums being involved per se, owing to the development of granulation tissue, and porosity of the dental process also, as shown by the accidental hard biting on one tooth, resulting in a permanent loosening of the same, due to an injury to the periodontium.

All seem to agree that the inflammatory conditions of the gums, either of local or systemic origin, act as a primary exciting cause in the production of alveolar atrophy. The irritating, inflamed and relaxed gums will sooner or later have a deleterious effect on the adjacent parts, namely, the periodontium and periosteum, inasmuch as it thereby affects the nutrition of the parts, and so damages the same, the influence being mostly felt in weakly developed, thin, paper-like septæ.

The pathology of these processes, no matter what the causes of the nutritional disturbances are, can be summed up in brief, as follows: Owing to the inflammatory process around about the alveolar border, which also influences the neighboring parts, the Haversian canals communicating with the periodontium and the outer periosteum become enlarged, the hyperemia of the gums with that of the periosteum, or periodontium, leads to a granulation of the medullary tissue in the canals, and so causes a light form of osteoporose to be developed. The dental process which in its normal condition has very small interstices, shows now much larger openings; the porosity which in its beginning is limited to the free border and slightly below, gradually extends deeper as more of the inflamed gum and its connecting periosteum are rendered unfit for supplying nutrition. The irritation also extending from the border to the periodontium, gives rise to the formation of periodontial granulations, owing to the granulations, which have principally sprung from the medullary tissue, the dental process becomes porous or rarified. The solution or wasting of the alveolar process, may proceed in a gradual way, as in cases of senile atrophy consuming many years, or where it occurs in association with some acute disease of the oral mucous membrane, and the gums run a rapid course, terminating in the loss of the teeth within a few days or weeks.

The state of the pockets varies as that of the gums themselves. They may be inflamed, reddened and swollen. Only in the minority of cases do we observe an apparent normal condition of the gums, and in the presence of the pockets find them closely fitting to the necks of the teeth so that a retention of deleterious substances is impossible. Few cases, however, remain in this condition; in the vast majority the pockets are not only deep and wide, but are patulous, so that foreign substances can enter easily and give rise to the development of bacteria and formation of pus, the presence of which has led many to believe it to be the cause of the disease, and gave rise to the coining of the name pyorrhea alveolaris by the French. The amount of secretion is dependent on the intensity of the inflammation, size of pocket, and to a great extent on the care and cleanliness bestowed upon the teeth and mouth by the of patient.

Pyorrhea alveolaris, then, is only a symptom and oftentimes wanting in the premature atrophy, although nearly all authors seem to think it to be the cause, or at least an essential requisite of the disease. The following facts, however, speak against it:

1st. In pronounced loosening and rapidly progressing alveolar atrophy there may not be present a trace pyorrhea.

2d. In light forms of atrophy and slight loosening of the teeth pyorrhea may be largely present.

3d. We have a gingival pyorrhea in children, resulting in the loosening of the teeth, but not accompanied by atrophy or absorption of the alveolar borders.

If a predisposition exists, it must be in the anatomical formation of the parts, and therefore is transmissible from parent to child, namely, a weak development and deficient nutrition of the alveolar process and roots of the teeth.

But to go into a full explanation of the so-called inherited tendencies would be beyond the scope of this paper, as it would necessitate a consideration of the minute anatomy, development and nutrition of the various parts concerned.

The treatment, as you are well aware, consists in the thorough removal of all deposits. This is easily said, but not so easily done, as it requires patience, time, and a delicate sense of touch, in order to detect the smallest particle on the root; and while removing the deposits, it is well at the same time to also remove by curetting the slightly necrotic portions of the alveolar ridge, being careful, however, not to mutilate the parts and thereby lessen the chance of recovery. For that purpose we formerly used the terrible looking Riggs' scalers, but we now have instruments much better suited for the removal of deposits in the delicate and well-shaped instruments of Drs. Harlan and Younger. After a thorough removal of the deposit and curettement of the pocket, removing the necrotic bone and granulation tissue, and washing out of the pockets by repeated syringing with hot water, apply lactic acid in a thorough manner, which application has a pronounced effect in promoting a healing of the affected parts, the lactic acid having not only the power of inducing a healthy granulation tissue to spring up, but thereby ensuring a union between the tissues of the pocket and the root, *i. e.*, by partly decalcifyiny the outer layers of the root, thus opening the mouths of the caniliculi, and stimulating the adjacent tissue to a healthy action.

Nothing accomplishes this so well as lactic acid and phenolsulphoricini, which action is well shown in its power of promoting a healthy granulation in tubercular ulcerations in the upper air passages, these being about the only remedies besides the application of galvanic current which will induce such ulcerations to heal. These remedies are best applied by soaking strands of twisted cotton in them, and then carefully packing into the pockets around the root, and leaving it in position for awhile, when you will find that the blood oozing out will be of a blackish color. After having pursued the above treatment, the patient is prescribed an antiseptic mouth bath, such as borolyptol, benzolyptus, or tar water and witch hazel, with hydronapthol and oil of cinnamon.

The above to be held in the mouth not less than three or four minutes and used thus frequently the first day; after that, in same manner three or four times daily; with careful toilet of the mouth.

Whenever I can induce the patient to take such care as to keep off the soft tartar which forms so readily in many of these cases, I feel confident of success, except where there has been a complete loss of the alveolar structures about the tooth, the same rising in the socket on the release of pressure; these latter being typical cases of pyorrhea alveolaris which have been allowed to advance too far, and should have been properly treated years before; yet even in some of these apparently hopeless cases a decided and often a lasting benefit will result from the proper treatment.

Besides the above local treatment it is well in the more marked cases to proscribe the too frequent use or indulgence of starchy foods, sugars and meats, advising the liberal use of water, also taking lithia salts in some form. Of late, the ozonized lithia water has been brought to my notice, and seems to be of benefit in cases where indicated. The cutting down of starchy foods and sugar, as well as the too liberal use of meats and heavy alcoholic beverages has, in my observation, been of decided benefit to these patients. At the same time I insist where I suspect an underlying constitutional trouble that they consult some medical practitioner regarding their case, and especially where, after a few thorough local treatments, I fail to get the proper response (or results), and have frequently observed that my local treatment in conjunction with the general treatment instituted by the medical practitioner would be decidedly more effectual and permanent. This is well marked in the gouty and rheumatic cases, and especially in diabetes mellitus; and here I might refer in an off-hand way to the rather distinct effect this disease has on the oral mucous membrane in its first affecting the superior maxilla, also in producing a distinct and characteristic odor in the mouth rather hard to describe, but once noticed easily recognized thereafter, (described best in German as "ein faden geruch."). In fact, the various symptoms produced might be looked upon as pathognomonic of the trouble, and should be carefully considered. The relationship of the oral manifestations in diabetes mellitus has been well shown by the investigations of Dr. F. Schneider Hofzahnarzt in Erlangen, and from whose article on this subject I have quoted.

In order to show and give you some idea as to these pathological processes in the bone (alveolar process) I have brought with me some micro-photographs made by my friend Dr. M. H. Fletcher, of Cincinnati, whose original and careful work in dental histology and pathology we ought all to commend. These micro-photographs will, I think, also prove beyond a doubt the existence of haversian canals in the alveolar process, a fact denied by some memcers of our society (The Odontological Society of Cincinnati) at the last meeting, when exceptions were taken to the statements of Dr. Fletcher and myself regarding this fact of their presence, and the influence exerted and part played by them in the repair and absorption of the alveolar process.

DISCUSSION.

Dr. Otto Arnold: It was said many centuries ago by a celebrated man, "If only my enemy would write a book." It is hard to write, but easy to criticise. It struck me very forcibly that it might be a good plan to make a parallel comparison between the theory of the two men, Dr. Baume and Prof. Peirce. Dr. Baume's theory was given in full in Dr. Heise's paper, and does not differ materially from Dr. Peirce's theory except that he opposes the term Pyorrhea Alveolaris and offers this one, "Atrophia Alveolaris Præcox." That term in plain English means simply premature atrophy of alveolus, etc. Now, as briefly as possible, I will give you Dr. Peirce's theory: "Pyorrhea alveolaris" is a generic term which, strictly defined, means a flowing of pus from an alveolus. Many terms have been given to this disease, but all simply represent as many diverse views. As the term is now understood, pyorrhea alveolaris includes all of those cases of morbid action characterized by the following features: A molecular necrosis of the retentive structures of the teeth (their ligament, the pericementum), an atrophy of the alveolar walls, together with a chronic hyperemia of the gum tissue which leads to limited hypertrophy. Also with a deposit of calculi upon their surfaces. The disease is generally but not always attended by the flow of pus.

Clinically, the cases in which these phenomena are observed may be divided into two classes: 1. Those in which the diseased process appears to begin at the gum margin. 2. Those in which the diseased process begins at some portion of the alveolus between the unbroken and apparently healthy gum margin and the apex of the root, the pulp of the tooth being alive.

These two case are so different that each requires a separate description. The first: The predisposing causes may all be included under the head of disorders causing a subacute inflammation of the gingivæ, accumulations on smokers' teeth, fermenting deposits of food, mouth-breathers' gingivitis, and mal-occlusion, etc. The exciting causes, however, are subgingival scaly deposits of calculi. This causes irritation of the gums which becomes more marked as the scaly deposits encroach upon the margins of the alveolus. The disease progresses, the teeth loosen, and ultimately drop out and the injected gum remains as a flabby mass, all disease ceases with the loss of the teeth. The process may involve any number of teeth.

The second class of pyorrhea seems to be but a local expression of the gouty diathesis and directly dependent on the deposition of the uric acid, urates and calcium salts in the pericemental membrane. The teeth in all such cases are almost exempt from caries.

The focus of the diseased action is confined to the region toward the apical extremity of the root. First there is a distinct swelling near the apex of the root, tooth becomes sensitive, and if we examine there will be found a distinct deposit upon the root, near the apex. As a result from a continual irritation from this deposit, there is inflammation which will extend, a disturbed relation between the blood and surrounding tissue increases, the gums become flaccid, spongy, altered in color and liable to hemorrhagic discharges; there is a gradual softening and absorption of the alveolar process. This is the theory advanced by Dr. Peirce. Now, this is, to my mind at least, a clearer and more definite etiology of this disease than is given by Prof. Baume. We have all seen these two kinds of disease, and we know from clinical experience that it is true.

I wish here to simply express myself and take exception to what this esayist has said about dentists not recognizing pyorrhea alveolaris, unless pus is present. I know it is generally supposed, or often at least, that dentists do not recognize it unless they see pus. While this is true to a certain extent, yet in the expression of the term as we have it to-day, we apply it more properly, therefore, I say, we do recognize a diseased condition there, oftentimes without the appearance of pus.

Now, in reference to its treatment, it is just about the same as all others have advocated in that line. Consists in a combination of local manipulations, some local surgery sometimes, and, of course, the main aim is to remove all the deposits from the roots.

The deposits which have been found on the roots where there are systemic disorders, these deposits have been removed and examined. They are found to consist of calcium and sodium urates, free uric acids and calcium phosphates. In the face of such decided, emphatic and definite experiments, I can hardly conceive how we can omit that cause from our consideration. Of course that means again in the treatment of these troubles that there must be some systemic treatment. It has been my custom for some time, when I find trouble of this kind, to inquire as to past history of the patient—if they ever had any rheumatic troubles or any symptoms of such. In this way we are sometimes able to establish a correct diagnosis as to their trouble and refer them for some systemic treatment. A marked case of this kind occurred in my own practice within the last half year. I will simply refer to it in this connection. I recognized the characteristic conditions as caused by this systemic pyorrhea alveolaris—no pus present, but all the evidences of the disease in the alveolus. I stated to the lady I would endeavor to do all I could for her. I asked as to her past health—if she had any rheumatic troubles. Why, she said, another doctor asked me the same question. I asked for what reason. I went to a doctor to have my eyes treated. I said so you had your eyes treated. For what trouble? The doctor said I had a very puzzling case. He said he had never seen anything like it; there was a deposit on the cornea of some substance which was preventing my regular vision. The doctor could do nothing for it and it got worse and worse.

Now, that is a very singular instance. It was undoubtedly a deposit of this same composition as we have in this pyorrhea—a uric acic sediment of some kind. It was to me a very interesting case and almost established beyond a doubt as to this class of deposits.

But now for the constitutional treatment. After we have arrested the trouble which is present, remove all deposits, for in a short time there will seem to be a marked improvement. But how long is that going to continue? Simply as long as the diathesis remains, and that of course we cannot control. Many treatments have been mentioned in the paper, lithia water perhaps having the greatest use. I have tried in addition to that and have recommended a number of times a tonic in the form of some of the salts of gold—gold bromide for example. There is a number of preparations of gold which seem to be doing a great deal of good just now. I have at least been able to report on patients changes in their constitutional habits and conditions by the use of some of these tonics. Whenever there has been a strong and marked tendency of systemic conditions I refer them to some physician. But I have recommended some preparations such as lithia water.

I agree again with the essayist in the statement that this is about the worst trouble we have to deal with in our practice and work.

Dr. H. A. Smith: I want to express my congratulations for this paper. Dr. Heise has made quite a study of this subject, and, being a German scholar, he has been able to read all articles on the latest work in that line and study it. We are inclined to accept as authority what he says in reference to this in Cincinnati. Dr. Witzell has called attention to a wasting away of the alveolus. He claims we have in the beginning a wasting of the border, which assumes a peculiar hollowed-out, gutter-like form, and which is characteristic of pyorrhea alveolaris. If this is true, it would be a means of diagnosing. Dr. Baume is about parallel with Witzell. Reference was made here to later ideas in reference to one phase of the disease, that formulated by Prof. Peirce. He and his school are enthusiastic in its treatment. Time is a great element in the treatment of pathological conditions, and, although we cannot agree with Dr. Peirce, we should at least respect that school and await further developments before saying they are entirely wrong. Dr. Heise said nothing about the idea that pyorrhea alveolaris is an expression of gouty diathesis. I have studied that phase of the subject and am not convinced, but I may state that we do have symptoms which resemble gout. Where we have no manifestion at the gum border and have this disease, it may be due to uric acid deposits. I have had these manifestations in my own teeth. It raises the question now whether we might not well investigate that subject. If we consider the teeth as set in a joint, and it is to my judgment simply a peculiar joint, the tooth is not fixed but movable, why may we not have deposits in this particular joint, uric acid deposits, as well as in any other joint, and that is significant of gout. If we have these expressions of pain in the periosteum, or what we call a sore tooth and neuralgia, why not say well, it is a joint, and perhaps we may have a deposit in the joint, and especially if we can learn the history from the patient. I am watching for the studying of this phase of pyorrhea from a gouty standpoint.

DR. J. R. CALLAHAN: I have just a word to say. I do not know a great deal about pyorrhea alveolaris. I am acquainted with Dr. Heise and his theory and am inclined to accept whatever he says in the line of treatment or diagnosis, or cause of pyorrhea, or anything of that nature.

He spoke in the latter part of his paper about Haversian canals and produced photographs by Dr. Fletcher. I should hesitate to criticise any statement Dr. Fletcher might make, but as far as the photographs are concerned I would like to have some gentleman point out to me the first indication of a Haversian canal in those photographs.

Dr. W. V. B. Ames, of Chicago: You have been extremely kind here to-day in making me one of your number, and I want to express my appreciation of it. The atmosphere seems to fit me a little better down here than any other part of the globe, and I thank you very much for the honor conferred upon me. This subject of pyorrhea alveolaris is extremely interesting to me and I want to say that this paper is the most resourceful paper I have ever heard on this subject; there is more in it and more to study, and from which we can get more valuable points, than any other paper I have ever heard read. I have just a few practical ideas to express. I believe these cases of difference cited are dependent upon each other. If we have neglected the teeth and there is a certain amount of deposit present, there comes a change in the systemic condition, with a breaking down, which is much aggravated by the local condition, and when we have present the systemic condition the condition of the gums will be in such a state that a local condition will be much more rapid.

Lactic acid treatment, I believe, is beneficial, and yet I think there is a virtue in the treatment in the use of gold and silver salts. Now, these are objectionable to some extent owing to their discoloring effect on teeth, yet I believe there is a great deal in it It is well known that a root discolored by silver-nitrate seems less likely to take on deposits afterward. These salts also have a general systemic beneficial effect. In the treatment of a root with silver-nitrate we get a surface on which calcareous deposits are less likely to adhere, but we also get a difference in the nature of the surface of the root. I have been using gold chlorid for a couple of years in the place of silver-nitrate; the latter in a deep pocket is decidedly irritating and may cause inflammation, while with gold chlorid we get the same effect without the irritation. I do not object to the discoloration of gold chlorid, and with two years' use I am justified in recommending it. I believe in the use of lithia water, and I have made some use of the gold chlorid solution tonics, dilute solutions.

The matter of retaining the same conditions in the treatment of pyorrhea is a matter of eternal vigilance; patient must return every three or six months, and if the patient can be worked up to the point of consulting a specialist, he will undergo more work and spend more money and you will accomplish better work.

I have here an instrument which I have had recently devised and will pass it about; it is a very good scaler, and seems to come about as near being perfect as any I have ever seen.

Dr. Heise: I do not know that I have anything special to say. I did refer to the gouty conditions as well as all the other forms. I want to say that this paper is not complete by any means, but is simply an outline to show that there is a phase of this subject which has not been touched upon to any extent except abroad.

In regard to Dr. Callahan's statement in reference to Haversian canals, it has nothing particular to do with the subject in question. He knows they are there, and if he cannot take the photograph as proof I am sorry.

I have used silver salts and lactic acid, a combination of the two. We do not get discoloration here. The use of the salts alone also close up the tubuli, and that is what we do not want. They seem to have the tendency of preventing the deposition of tartar around the roots of the teeth and keep the teeth cleaner. I could not go into detail with everything, for I would have had to make a small book out of it.

Dr. Taft: Do not lactic acid and silver salts affect the calcareous material?

Dr. Heise: They alone have strengthening effects upon the teeth, but they close up the tubuli, and I think there is a regrowth of the periosteum about the root. With lactic acid and the salts we also get a tendency to prevent deposition of calcic salts.

Dr. Taft: Will not silver-nitrate close the tubuli more definitely than lactic acid?

Dr. Heise: Yes, but all the salts do this, but the lactic acid salts less than the others. I have never tried the gold salts, but I will try them.

Dr. Taft: Well, with the use of the acids with the salts we get a deposition of the salt, of course; we have a nitrate deposit on the surface, but it is not as penetrating as the nitrate of silver alone. Does it not make a difference in the treatment, whether the case is of long standing or recent?

Dr. Heise: Yes, in every instance; a man has to use his own judgment. There is a difference in the treatment of these two cases. If we get a complete loss of tissue around the teeth, I think the best thing to do is to extract the tooth. We may be able to help it for a time by the correct treatment, but it will go back to the original condition again. Success in the treatment of this trouble, like that in many others, lies in the early recognition of the disease. We cannot accomplish a cure by the mere removal of the tartar from the roots. Dr. Ames' instruments are about as good as any I have seen. The benefit of the Younger scaler lies in the fact that it does not scratch the root of the tooth. We should not cut the root at all.

Ques.: Is it necessary to be careful not to lacerate the tissue and cause hemorrhage, or is it better to have a free hemorrhage?

Dr. Heise: Never lacerate the tissue any more than is necessary, as it only lengthens the time of healing.

Dr. Junkerman: How do you differentiate between a local trouble and a systemic trouble?

Dr. Heise: Of course there is a decided difference between the two; although to a certain extent one is dependent on the other. I am aware that the great variety of statements made by different men, Dr. Peirce and others have greatly misled every one. My idea in the paper was not to bring out the different theories, but to go into consideration of this man's, for that and the other would take more time than is at our disposal.

